# An Open-Structure Treadmill Gait Trainer: From Research to Application

**DOI:** 10.1155/2017/9053630

**Published:** 2017-06-15

**Authors:** Jian Li, Diansheng Chen, Yubo Fan

**Affiliations:** ^1^Beijing Key Laboratory of Rehabilitation Technical Aids for Old-Age and Disability and Key Laboratory of Rehabilitation Aids Technology and System of the Ministry of Civil Affairs and Engineering Research Center for Rehabilitation Aids of the Ministry of Civil Affairs, National Research Center for Rehabilitation Technical Aids, Beijing 100176, China; ^2^Robotic Institute, Beihang University, Beijing 100191, China; ^3^Key Laboratory for Biomechanics and Mechanobiology of Ministry of Education, School of Biological Science and Medical Engineering, Beihang University, Beijing 100191, China

## Abstract

Lower limb rehabilitation robots are designed to enhance gait function in individuals with motor impairments. Although numerous rehabilitation robots have been developed, only few of these robots have been used in practical health care, particularly in China. The objective of this study is to construct a lower limb rehabilitation robot and bridge the gap between research and application. Open structure to facilitate practical application was created for the whole robot. Three typical movement patterns of a single leg were adopted in designing the exoskeletons, and force models for patient training were established and analyzed under three different conditions, respectively, and then a control system and security strategy were introduced. After establishing the robot, a preliminary experiment on the actual use of a prototype by patients was conducted to validate the functionality of the robot. The experiment showed that different patients and stages displayed different performances, and results on the trend variations across patients and across stages confirmed the validity of the robot and suggested that the design may lead to a system that could be successful in the treatment of patients with walking disorders in China. Furthermore, this study could provide a reference for a similar application design.

## 1. Introduction

Lower limb rehabilitation robots have been actively investigated for the past two decades [1, 2], with a number of studies showing the significance of these robots in the rehabilitation of patients with lower limb dysfunctions [3], such as stroke, central nervous system disorders, spinal cord injuries [4], and cerebrovascular diseases [5]. Since the last century, many scientists and research institutes in different countries have developed various rehabilitation robots [6, 7]. However, many of these rehabilitation robots were confined in research laboratories and were not used in practical health care, particularly in China. According to Díaz et al. [8], these robot systems can be classified into five types: (i) treadmill gait trainers, (ii) foot-plate-based gait trainers, (iii) overground gait trainers, (iv) stationary gait trainers, and (v) ankle rehabilitation systems. The only commercialized lower limb rehabilitation robots were Lokomat [9], LokoHelp, ReoAmbulator, Gangtrainer GT [10], ReWalk [11, 12], and HAL [13]. Generally, the following questions had yet to be answered: What limits the application of rehabilitation robots? What is the gap between robots and patients? Previous studies always found the problems in technology, but progress in solving these problems had been minimal. Although technical realization is the basis for the robot using, it is not the only element for a truly viable product, in which availability and convenience of design are indispensable to the success of a product in practice.

Unquestionably, China has the biggest population and elderly population as well as the most number of patients with lower limb dysfunctions among the countries in the world. Currently, only few individuals in the country can afford long-term treatments and expensive fees of rehabilitation robots which were almost imported from foreign countries [14]. Furthermore, the payment schemes in China further hinder the purchase of these robots [15, 16]. At present, a rehabilitation robot was not included in the medicare, and only disabled soldiers, praise persons, and work-injury persons can share some fees by the medicare. Although researchers could establish a technique for rehabilitation robots, application feasibility of these robots remains uncertain. Therefore, development of a low-cost and easy-to-use lower limb rehabilitation robot is imperative in China. Accordingly, Tsinghua University [17], the Harbin Institute of Technology, Shanghai Jiao Tong University, and Zhejiang University, among others [18], have tried to address this need and developed more than 20 models of rehabilitation robots. However, these robots also have not come into application because of many reasons [19]. There are also lots of problems existing on the application. For example, some robots used heavy blocks and pulleys for the design of a suspension device, and these components inevitably increased the scale of the robot; some robot's exoskeletons were installed with handrails and other components that would hinder the patients to wear or remove the exoskeletons [20, 21]; and some robot's adjusting devices were complicated, such as armrests and exoskeletons of Lokomat could be adjusted, respectively, including width and height [9], but the adjustments were time consuming in application; in addition, the considerably long preparation time for robots often led to an off-putting feeling to the patients [1]. Therefore, exoskeletons that are easy and quick to wear and remove and those that allow for comfortable and healthy rehabilitation conditions should be developed [2].

Accordingly, the objective of this study is to introduce and design an open-structure and applicable treadmill gait trainer with features such as a simple structure and control scheme, low cost, and a reliable security. The design was developed with the key aims of providing good application that is both simple and convenient. In the next sections, important mechanism design and control scheme of the robot are presented; this will be followed by a description of prototype testing and analyses of the outcomes.

## 2. Materials and Methods

In order to meet rehabilitation needs of people with lower limb dysfunction in China and provide good application for rehabilitation robot, an open-structure treadmill gait trainer with features of easy-to-use structure, high security, and cost-effectiveness was aimed to be created. The robot was schemed to solve practical problems for patients, such as difficulty on wearing exoskeleton and long preparation time.

### 2.1. Device Description

**Figure 1:**
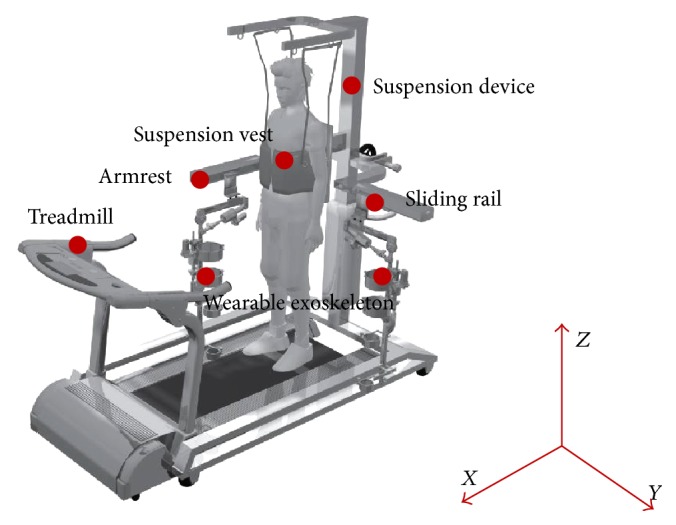
Diagram of an open-structure design for the robot and key components.

As shown in Figure 1, this open-structure concept was influenced by looking at problems of existing related robots, thinking about why related robots often use parallel rods (relatively closed structure) as armrests and why the exoskeletons were fixed, which would prevent patients from wearing or removing exoskeletons and getting on and off treadmills. In this study, each armrest could open and rotate 90° to allow a patient to easily move his or her wheelchair up and down the treadmill and wear the suspension vest. Under the armrests, there were two sliding rails. The exoskeleton can slide back and forth; as a result, it was easy to wear. The core structure of the exoskeleton was planned to manufacture with magnesium alloy AZ61, which is the lightest in the practical metals. Besides, the width and height of armrests and exoskeletons could be adjusted, respectively. It was considered that this principle of open structure could be applied to make a patient's training convenient and less-time preparation. The robot (treadmill gait trainer) consists of a wearable exoskeleton, suspension vest, suspension device, treadmill, and so on. Among them, a wearable exoskeleton, suspension device, and control system were the most important parts, which were described as follows:

#### 2.1.1. Wearable Exoskeleton

**Figure 2:**
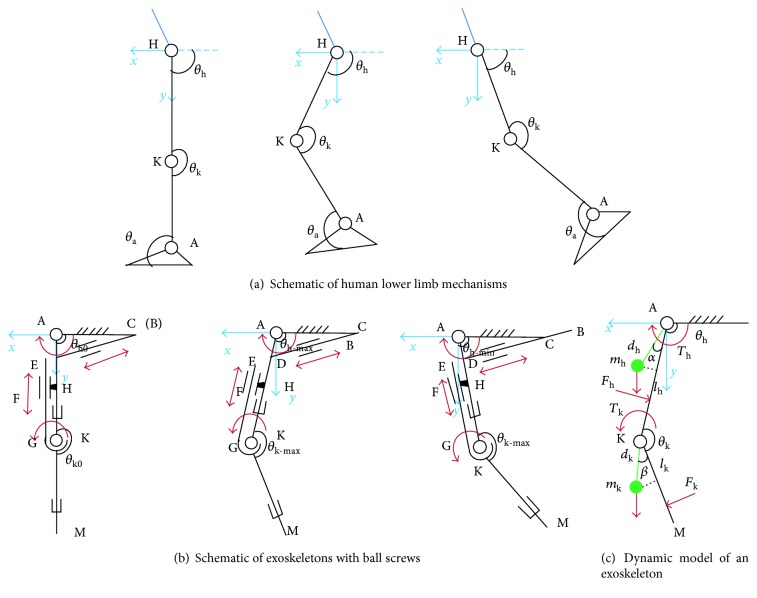
Analysis and design of exoskeleton: hip joint was the origin of the coordinate system, forward direction was the *x*-axis, and downward direction was the *y*-axis.

Human locomotion is a rhythmic task: periodic, coordinated, and balanced, which is divided into stance phase and swing phase. And the lower limb usually displays complicated three-dimensional movement on sagittal plane, coronal plane, and horizontal plane in daily life. However, in rehabilitation training, gait on the coronal plane is usually disregarded [22]. In order to analyze hip and knee motion and provide real-time optical data for the exoskeleton design, Vicon motion capture system (Oxford Metrics Limited Company, UK) was used in this study. As shown in Figure 2(a), it was found that the movement of a single leg from stance to walk follows three typical patterns. In the initial state, the lower limb keeps in a straight line. Assuming the hip joint was the origin of the coordinate system, the angles of the hip and knee were 90° and 180°, respectively. In the forward-swing state, the angle of the hip becomes larger than 90°, and the knee angle becomes smaller than 180°. In the rear-swing state, both the hip and knee angles become smaller [23].

On the basis of the above analysis [24], a schematic of the proposed exoskeleton was conceived in Figure 2(b), in which “A” was the hip joint, “K” was the knee joint, and “M” was the ankle joint. Similarly, coordinates of “A”, “K”, and “M” were (0, 0, 0), (*x*_K_, *y*_K_, 0), and (*x*_M_, *y*_M_, 0), respectively, and could be expressed as

(1) $$\begin{array}{c} \begin{array}{c} x_{K}=l_{AK}\cos\left(180^{\circ }-\theta_{h}\right)\\ y_{K}=l_{AK}\sin\left(180^{\circ }-\theta_{h}\right)\\ Z_{K}=0. \end{array} \end{array}$$

(2) $$\begin{array}{c} \begin{array}{c} x_{M}=l_{AK}\cos\left(180^{\circ }-\theta_{h}\right)-l_{KM}\sin\left(270^{\circ }-\theta_{k}-\theta_{h}\right)\\ y_{M}=l_{AK}\sin\left(180^{\circ }-\theta_{h}\right)+l_{KM}\cos\left(270^{\circ }-\theta_{k}-\theta_{h}\right)\\ Z_{M}=0. \end{array} \end{array}$$

In the initial state, *θ*_h_ was a right angle, whereas *θ*_k_ was a straight angle; thus, the coordinates of “K” and “M” could be simplified to

(3) $$\begin{array}{c} \begin{array}{c} x_{K}=0\\ y_{K}=l_{AK}\\ Z_{K}=0. \end{array} \end{array}$$

(4) $$\begin{array}{c} \begin{array}{c} x_{M}=0\\ y_{M}=l_{AK}+l_{KM}\\ Z_{M}=0. \end{array} \end{array}$$

In the forward-swing state, coordinates of “K” and “M” could also be expressed by (1) and (2). Given that *θ*_h_ was an obtuse angle, so

(5) $$\begin{array}{c} \cos\left(180^{\circ }-\theta_{h}\right)>0, \end{array}$$

and when

(6) $$\begin{array}{c} l_{AK}\cos\left(180^{\circ }-\theta_{h}\right)>l_{KM}\sin\left(270^{\circ }-\theta_{k}-\theta_{h}\right), \end{array}$$

then

(7) $$\begin{array}{c} x_{M}>0. \end{array}$$

Both “K” and “M” were on the left side of the *y*-axis, but when

(8) $$\begin{array}{c} l_{AK}\cos\left(180^{\circ }-\theta_{h}\right)<l_{KM}\sin\left(270^{\circ }-\theta_{k}-\theta_{h}\right), \end{array}$$

it would display

(9) $$\begin{array}{c} x_{M}<0. \end{array}$$

Perhaps, “K” was on the left side of the *y*-axis, but “M” was on the right side.

In the rear-swing state, given that *θ*_h_ was an acute angle, so

(10) $$\begin{array}{c} \cos\left(180^{\circ }-\theta_{h}\right)<0, \end{array}$$

and

(11) $$\begin{array}{c} x_{M}<0. \end{array}$$

Therefore, specific structures of the exoskeleton were designed with two sides, including the right exoskeleton and the left exoskeleton, which were consist with Figure 2(b). Each side had two joints for simulating the extension and flexion of the human hip and knee, and ball screws were used to create the structure to ensure safety. The lengths of thigh rods and crus rods could be adjusted to fit different individual needs. Moreover, weight of exoskeleton was kept to a minimum.

Furthermore, high-output torque ratios of drive mechanisms are crucial to actual use. Therefore, a dynamic model (see Figure 2(c)) [25, 26] was initially developed based on Lagrangian, where coordinates of the mass centers for the hip (*m*_*h*_) and knee (*m*_*k*_) were as follows:

(12) $$\begin{array}{c} \begin{array}{c} x_{m_{h}}=d_{h}\cos\left(180^{\circ }-\theta_{h}-\alpha \right)\\ y_{m_{h}}=d_{h}\sin\left(180^{\circ }-\theta_{h}-\alpha \right)\\ Z_{m_{h}}=0 \end{array}, \end{array}$$

(13) $$\begin{array}{c} \begin{array}{c} x_{m_{k}}=l_{AK}\cos\left(180^{\circ }-\theta_{h}\right)-d_{k}\sin\left(270^{\circ }-\theta_{k}-\theta_{h}-\beta \right)\\ y_{m_{k}}=l_{AK}\sin\left(180^{\circ }-\theta_{h}\right)+d_{k}cos\left(270^{\circ }-\theta_{k}-\theta_{h}-\beta \right)\\ z_{m_{k}}=0. \end{array} \end{array}$$

Therefore, the velocities of *m*_*h*_ and *m*_*k*_ were

(14) $$\begin{array}{c} \begin{array}{c} {\dot{x}}_{m_{h}}=d_{h}\sin\left(180^{\circ }-\theta_{h}-\alpha \right){\dot{\theta }}_{h}\\ {\dot{y}}_{m_{h}}=-d_{h}\cos\left(180^{\circ }-\theta_{h}-\alpha \right){\dot{\theta }}_{h}\\ {\dot{Z}}_{m_{h}}=0 \end{array} \end{array}$$

and

(15) $$\begin{array}{c} \begin{array}{c} {\dot{x}}_{m_{k}}=l_{AK}\sin\left(180^{\circ }-\theta_{h}\right){\dot{\theta }}_{h}-d_{k}\left({\dot{\theta }}_{k}+{\dot{\theta }}_{h}\right)\cos\left(270^{\circ }-\theta_{k}-\theta_{h}-\beta \right)\\ {\dot{y}}_{m_{k}}=-l_{AK}\cos\left(180^{\circ }-\theta_{h}\right){\dot{\theta }}_{h}+d_{k}\left({\dot{\theta }}_{k}+{\dot{\theta }}_{h}\right)\sin\left(270^{\circ }-\theta_{k}-\theta_{h}-\beta \right)\\ {\dot{z}}_{m_{k}}=0, \end{array} \end{array}$$

and the heights of *m*_*h*_ and *m*_*k*_ were

(16) $$\begin{array}{c} \begin{array}{c} h_{m_{k}}=l_{AK}\sin\left(180^{\circ }-\theta_{h}\right)+l_{KM}\cos\left(270^{\circ }-\theta_{k}-\theta_{h}\right)-d_{h}\sin\left(180^{\circ }-\theta_{h}-\alpha \right)\\ h_{m_{h}}=l_{KM}\cos\left(270^{\circ }-\theta_{k}-\theta_{h}\right)-d_{k}cos\left(270^{\circ }-\theta_{k}-\theta_{h}-\beta \right). \end{array} \end{array}$$

Given that the mass of the hip and knee consists of exoskeleton mass, user mass, actuator mass, and other component mass, total mass could be calculated as [27]

(17) $$\begin{array}{c} \begin{array}{c} m_{h}=m_{exo\;h}+m_{uerh\;h}+m_{act\;h}+m_{oth\;h}\\ m_{k}=m_{exo\;k}+m_{uerh\;k}+m_{act\;k}+m_{oth\;k}, \end{array} \end{array}$$

where *m*_exo h_ was the mass of the hip exoskeleton, *m*_uerh h_ was the mass of the user's hip, *m*_act h_ was the mass of the hip actuators, and *m*_oth h_ was the mass of the other hip components, such as fastening tape. Similarly, *m*_exo k_, *m*_uerh k_, *m*_act k_, and *m*_oth k_ were the mass of the knee exoskeleton, user's knee, knee actuators, and other knee components, respectively. Therefore, according to the Lagrangian formulations,

(18) $$\begin{array}{c} E_{k}=E_{k1}+E_{k2}=\frac{1}{2}m_{h}q_{1}^{2}+\frac{1}{2}m_{k}q_{2}^{2} \end{array}$$

and

(19) $$\begin{array}{c} \begin{array}{c} q_{1}^{2}={\dot{x}}_{m_{h}}^{2}+{\dot{y}}_{m_{h}}^{2}\\ q_{2}^{2}={\dot{x}}_{m_{k}}^{2}+{\dot{y}}_{m_{k}}^{2}. \end{array} \end{array}$$

Meanwhile,

(20) $$\begin{array}{c} E_{p}=E_{p1}+E_{p2}=m_{h}gh_{m_{h}}+m_{k}gh_{m_{k}} \end{array}$$

and

(21) $$\begin{array}{c} L=E_{k}-E_{p}. \end{array}$$

The hip joint torque and knee joint torque were

(22) $$\begin{array}{c} \begin{array}{c} T_{h}=f_{h}=\frac{d}{dt}\frac{\partial L}{\partial {\dot{q}}_{1}}-\frac{\partial L}{\partial q_{1}}\\ T_{k}=f_{k}=\frac{d}{dt}\frac{\partial L}{\partial {\dot{q}}_{2}}-\frac{\partial L}{\partial q_{2}}. \end{array} \end{array}$$

Taking into account the actual situation, an additional coefficient was considered to ensure the reliability of the design in order to avoid unexpectedly large loads. These considerations were expressed in (16).

(23) $$\begin{array}{c} \begin{array}{c} T_{h‐\max}=c_{h}T_{h}\\ T_{k‐\max}=c_{k}T_{k}, \end{array} \end{array}$$

where *c*_h_ and *c*_k_ were the coefficients for hip joint and knee joint and *c*_h_ > 1 and *c*_k_ > 1. Moreover, motor torque for the hip and knee also could be calculated by [28]

(24) $$\begin{array}{c} \begin{array}{c} T_{h‐mor}=\frac{T_{h‐\max}\Delta L_{h‐screw}}{2\pi r_{h‐bet}d_{h}}\\ T_{k‐mor}=\frac{T_{k‐\max}\Delta L_{k‐screw}}{2\pi r_{k‐bet}d_{k}}, \end{array} \end{array}$$

where *T*_h‐mor_ and *T*_k‐mor_ were the motor torques for the hip joint and knee joint, respectively; *r*_h‐bet_ and *r*_k‐bet_ were the ratios between the diameters of belt wheels, respectively, connected to the ball screws and the DC motors; and Δ*L*_h‐screw_ and Δ*L*_k‐screw_ were the leads of the ball screws for the hip joint and knee joint, respectively. Thus, hip and knee motors could be selected based on the values of *T*_h‐max_ and *T*_k‐max_. In addition, the output torque of the joint was determined by the force along the ball screws and the distance between the center of the joints' rotation and ball screws. Therefore, when transmission loss and mechanism mass were not considered, ratios of output torque for the hip joint and the knee joint could be formulated as follows [29]:

(25) $$\begin{array}{c} \begin{array}{c} r_{h}=\frac{T_{h‐\max}}{T_{h‐mor}}=\frac{2\pi r_{h‐bet}d_{h}}{\Delta L_{h‐screw}}\\ r_{k}=\frac{T_{k‐\max}}{T_{k‐mor}}=\frac{2\pi r_{k‐bet}d_{k}}{\Delta L_{k‐screw}}. \end{array} \end{array}$$

Here, *r*_h_ and *r*_k_ denoted the output torque ratios for the hip and knee joint, respectively, and *d*_h_ and *d*_k_ were the distances between the joints and ball screws, respectively. Both distances varied with the angles of the associated joint (*θ*_h_ and *θ*_k_). Furthermore, (25) suggested that a higher output torque ratio could be obtained for the DC motor by increasing *r*_k‐bet_, *r*_h‐bet_, *d*_k_, and *d*_h_ [30]. All of the above detailed adjustments were beneficial and important for the application design.

#### 2.1.2. Suspension Device

**Figure 3:**
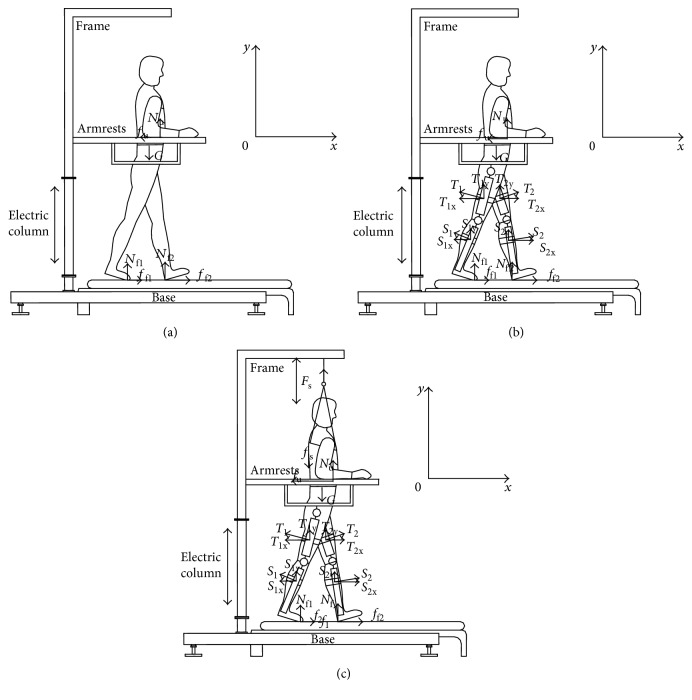
Force model for patient training. (a) Walking without exoskeletons and suspension device. (b) Walking with exoskeletons but without suspension device. (c) Walking with both exoskeletons and suspension device [32].

Generally, there are three situations that a patient normally encounters with treadmill-gait training. As illustrated in Figure 3, the sagittal axis and vertical axis of the body were defined as the *x* direction and *y* direction, respectively. When ∑*F*_x_ = 0 (*F*_x_ was the horizontal force), ∑*F*_y_ = 0 (*F*_y_ was the vertical force), and ∑*M* = 0 (*M* was the torque), the following equations were derived [31].

When the patient walked on the treadmill without exoskeletons and suspension device, the governing equations were

(26) $$\begin{array}{c} \begin{array}{c} 2f_{u}=f_{f1}+f_{f2}\\ 2N_{u}+N_{f1}+N_{f2}=G\\ N_{f1}l_{Nf1}=N_{f2}l_{Nf2}+N_{u}l_{Nu}+f_{f1}l_{f1}+f_{f2}l_{f2}+f_{u}l_{fu}. \end{array} \end{array}$$

When the patient walked on the treadmill with exoskeletons but without suspension device, the governing equations were

(27) $$\begin{array}{c} \begin{array}{c} T_{2x}+S_{2x}+f_{f1}+f_{f2}=T_{1x}+S_{1x}+2f_{u}\\ 2N_{u}+T_{1y}+T_{2y}+S_{1y}+S_{2y}+N_{f1}+N_{f2}=G\\ T_{1}l_{T1}+S_{1}l_{s1}+N_{f1}l_{Nf1}=T_{2}l_{T2}+S_{2}l_{s2}+N_{f2}l_{Nf2}+N_{u}l_{Nu}+f_{f1}l_{f1}+f_{f2}l_{f2}+f_{u}l_{fu}. \end{array} \end{array}$$

When the patient walked on the treadmill with both exoskeletons and suspension device, the governing equations were

(28) $$\begin{array}{c} \begin{array}{c} T_{2x}+S_{2x}+f_{f1}+f_{f2}=T_{1x}+S_{1x}+2f_{u}\\ F_{s}+2N_{u}+T_{1y}+T_{2y}+S_{1y}+S_{2y}+N_{f1}+N_{f2}=G+f_{s}\\ T_{1}l_{T1}+S_{1}l_{s1}+N_{f1}l_{Nf1}=T_{2}l_{T2}+S_{2}l_{s2}+N_{f2}l_{Nf2}+N_{u}l_{Nu}+f_{f1}l_{f1}+f_{f2}l_{f2}+f_{u}l_{fu}. \end{array} \end{array}$$

In (26), (27), and (28), *F*_s_ was the lifting force provided by the suspension device to the body; *f*_s_ was the friction force provided by the suspension vest to the body; *N*_u_ and *f*_u_ were the support force and horizontal friction force on the arm of the patient; *T*_1x_, *T*_2x_, *T*_1y_, and *T*_2y_ were the component forces exerted by the exoskeletons on the thigh in the *x* and *y* directions, respectively; *S*_1x_, *S*_2x_, *S*_1y_, and *S*_2y_ were the component forces exerted by the exoskeletons on the crus in the *x* and *y* directions, respectively; and *f*_f1_, *f*_f2_, *N*_f1_, and *N*_f2_ were the normal forces and static friction forces for the two feet.

As shown in Figure 3(a) and suggested by (26), when a patient walked on the treadmill without exoskeletons and a suspension device, entire body weight was concentrated on the legs and arms, and the balance of the body was entirely controlled by the hands and feet. If the legs lose support capacity, both arms of the patient would be subjected to discomfort feeling because of a larger support force and balance force. When the patient walked on the treadmill with exoskeletons but not including a suspension device, as indicated in Figure 3(b) and (27), the exoskeleton could exert a certain amount of support force to the body, thereby reducing support force from the legs. However, support force from the arms and the balance force were not significantly reduced. When the patient walked on the treadmill both with the exoskeletons and suspension device, as indicated in Figure 3(c) and (28), drastic changes occur. The suspension device provided lifting force *F*_s_ to the body, and both forces on the legs and arms were reduced; as a result, the patient would feel comfortable. However, *F*_s_ could also introduce *f*_s_. When *F*_s_ increases, the forces on the legs and arms were reduced, but *f*_s_ increased; consequently, the suspension vest would slide into the armpit. Given that numerous blood vessels, nerves, and lymph nodes are located in the armpit region, prolonged impingement in this region could cause the upper limb to feel numb. At the same time, *T*_1x_, *T*_2x_, *S*_1x_, *S*_2x_, *N*_u_, *f*_u_, *T*_1y_, *T*_2y_, *S*_1y_, and *S*_2y_ also had direct influences on body comfort. Nevertheless, the forces on the arm were reduced; thus, the influences of *N*_u_ and *f*_u_ were negligible, and *T*_1x_, *T*_2x_, *S*_1x_, *S*_2x_, *T*_1y_, *T*_2y_, *S*_1y_, and *S*_2y_ were relatively stable for one person with fastening tapes. If the exoskeletons were sufficiently light and the degree of tightness between the lower limbs and exoskeletons was appropriate, the patient would be comfortable during training because of good coordination [33].

#### 2.1.3. Security Strategy

**Figure 4:**
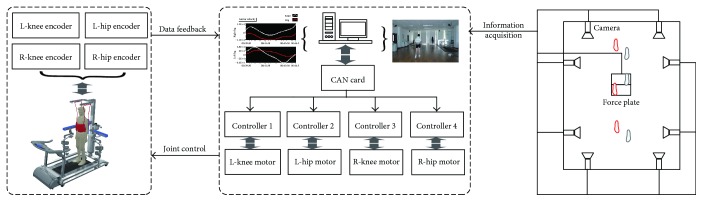
Architecture of control system and logic.

As shown in Figure 4, a closed-loop control scheme was designed [34], the standard information on the hip and knee were obtained with Vicon motion capture system and stored in a computer. The industrial computer served as the master controlling the four motors on both sides of the exoskeletons via a controller area network bus; subsequently, the encoders relayed the position, angle, velocity, and torque of the motor to the computer [35]. Besides, different human-machine parameters (50th percentile and 5th percentile) and training modes (time mode and step mode) were considered and designed to avoid joints exceeding the setting angles and incurring secondary damage.

**Figure 5:**
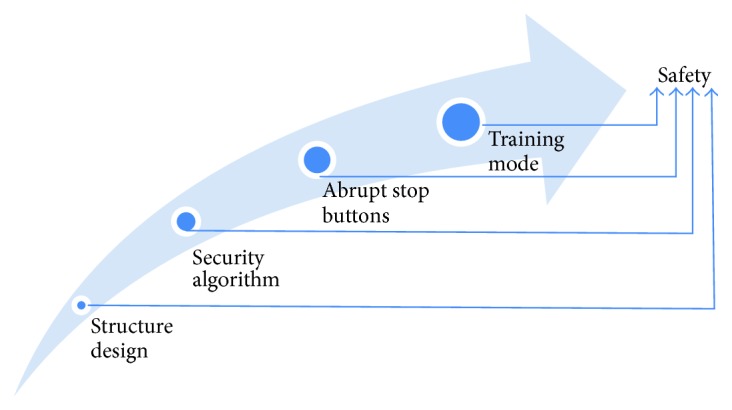
Security strategy for the robot.

Figure 5 shows that certain considerations were emphasized in the mechanical design and control design for practical application [36]. Firstly, ball screws were used at the hip and knee joints, and the itinerary of the ball screws were accurately calculated to limit the rotation range and prevent reverse rotation. This safeguard comprised the last line of defense against mishaps, providing protection even when other security protocols failed to take effect. Secondly, as an intelligent protection, a security algorithm for the control system was established. When the encoders detected that the position, angle, velocity, and torque values of the joints exceeded the scope of protection, drive system would be interrupted, offering the fastest security control. Thirdly, an abrupt stop button was installed on the training platform, and rehabilitation physicians could immediately cut off the power supply. Finally, time mode was designed based on the time setting, and the robot could be stop by clicking the “STOP” icon on the computer screen. All the aforementioned four security protocols were independent. Thus, the robot could not possibly cause or incur accidental damages even when some of the protocols were invalid, unless all four security protocols were breached at the same time. However, this situation was extremely rare.

### 2.2. Prototype Testing

**Figure 6:**
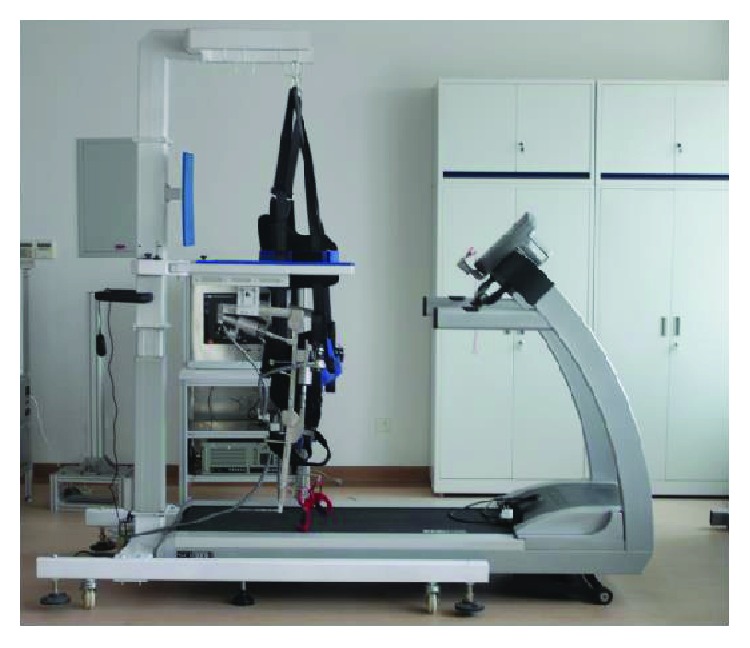
Prototype of the robot.

After finalizing the detailed design and analysis of a treadmill gait trainer, a prototype (Figure 6) was processed and assembled [37, 38], whose parameters were listed in Table 1.

In order to validate the functionality of the robot, a prototype experiment was conducted, which involved four patients (males aged at 38, 42, 30, and 46, resp.; mean age 39; mean weight 62 kg; mean height 163 cm). It was noted that only patients with a clear mind but with different degrees of walking disorders were selected for the experiment. All ethical issues with the experiment was in accordance with the local ethical regulations and approved by the institutional review boards, and all of the subjects consensually participated. After signing a protocol, they were trained twice every day for ten days completely free of charge under the care of two doctors. Each person was lifted until the feet would leave the treadmill, and every training session lasted 20 minutes.

## 3. Results and Discussion

After ten days of training, all of the patients found that the robot prototype was acceptable. Patients and doctors alike provided many recommendations, which were very useful for the future improvement. Moreover, sizeable amounts of data were obtained, such as joint position, speed, and torque, which could evaluate the availability directly, in particular joint torque.

### 3.1. Analysis on Different Patients

**Figure 7:**
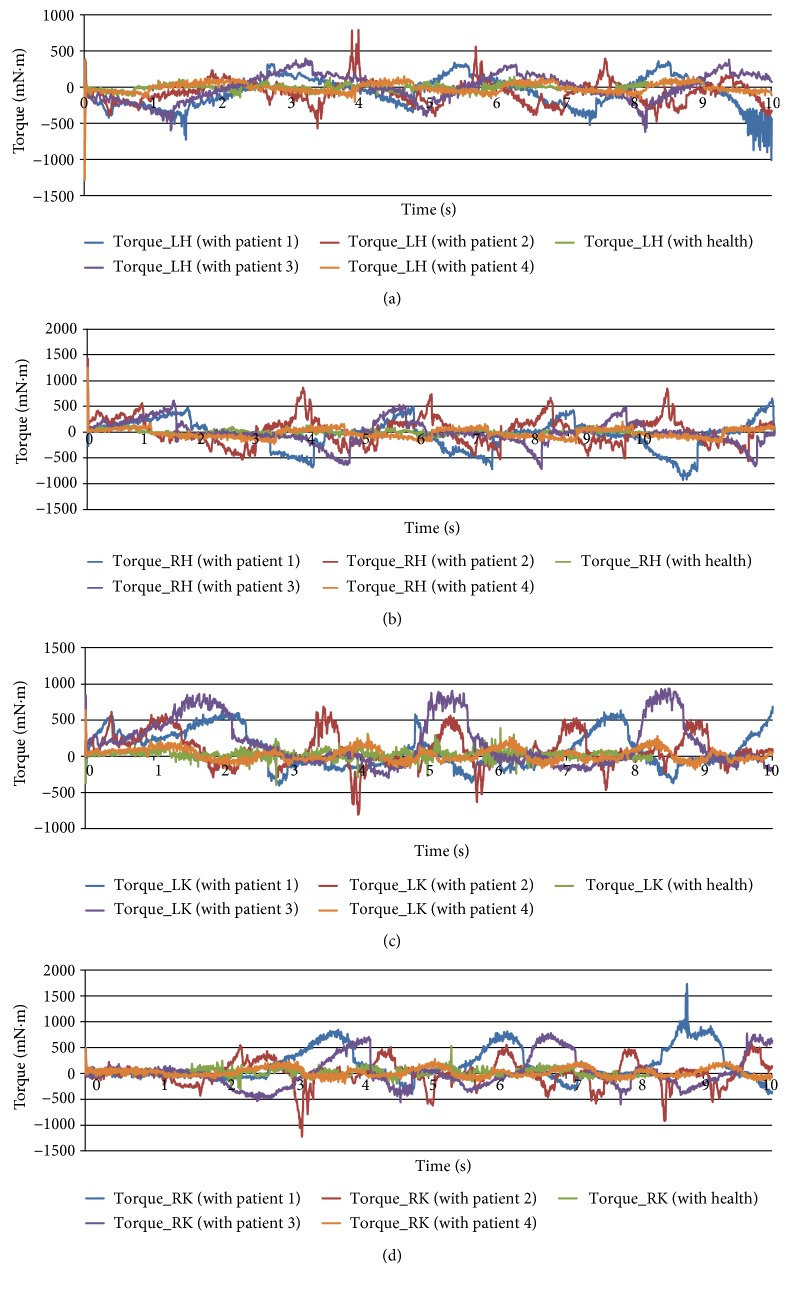
Joint torques on different patients. (a) Left hip. (b) Right hip. (c) Left knee. (d) Right knee.

As shown in Figure 7, in the case of the same degree of weight loss, joint torques and the time of gait cycle varied across patients because of differences in body sizes, pathological conditions, and other factors, but variation trends were similar and consistent with each other. This phenomenon showed that the robot was sensitive to differences among patients. Moreover, torque requirements of joint motors showed a certain degree of diversity, and the maximum torque values for the hip joint (Figures 7(a) and 7(b)) of patient 1 (P1), patient 2 (P2), and patient 3 (P3) could be higher than that of patient 4 (P4) and demonstrated large fluctuations. By contrast with knee torques (Figures 7(c) and 7(d)), the maximum torque in the torque values of P4 were always minimal in the four patients, whose fluctuations values were relatively stable. However, all of the data on the left hip, right hip, left knee, and right knee of P4 showed remarkable bigger values of no load. Nevertheless, some unusual data on the patients are obtained. On the basis of engineering experience, it could suspect that these deviations are due to the interaction between human and robot. When the human was scared or nervous, the machine may demonstrate poor trackability; as a result, outliers appear. This was in line with the actual situation.

**Figure 8:**
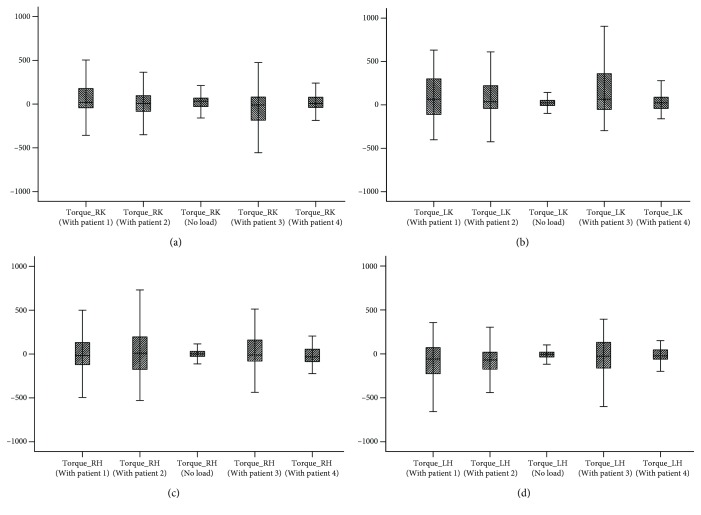
Statistical analysis of torque on different patients. (a) Left hip. (b) Right hip. (c) Left knee. (d) Right knee.

In addition, statistical analysis of joint torques was conducted to further understand the joint torque requirements of different patients [39]. The pictures (Figure 8) illustrated the maximum, minimum, median, and 25th and 75th percentiles of the motor torque for the hip joints and that for the knee joints. For the left hip (Figure 8(a)), P4 demonstrated the most concentrated torque which had the smallest value, whereas P1 and P3 showed the most dispersed torque and the largest value. For the right hip (Figure 8(b)), P4 again presented the most concentrated torque and the smallest value, whereas P2 presented the most dispersed torque and the largest value. A comparison of the other torques are found in Figures 7(c) and 7(d), which show that P3 always had the most dispersed torque and the largest value. However, P4 was just the opposite. Based on the analysis of Figure 7 and the above results, it could be inferred that the illness of P4 would be lighter, whose lower limb may lose part of walking function but not completely, and P3 may had a serious problem. Finally, it was found that these results were consistent with the pathological conditions, in which P4 could walk slowly but unstably and short term and P4 was unable to walk completely by himself. This finding confirmed that the robot was credible in practice, and performance of the patient with it was consistent with the actual conditions.

### 3.2. Analysis on Different Stages

**Figure 9:**
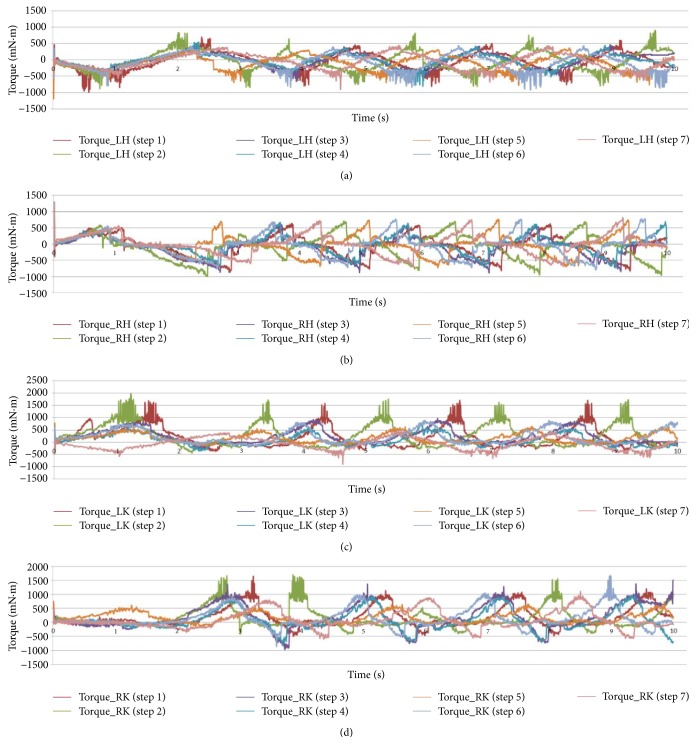
Experimental data of one patient at different stages. (a) Left hip. (b) Right hip. (c) Left knee. (d) Right knee.

Furthermore, different stages for one patient were showed in Figure 9 [40]. Torque variations for a patient at different stages were different but had very similar trends. However, in stages 1 and 2, numerous noise points existed, causing the overall curves to fluctuate repeatedly (except for Torque_RH). The noise points were understandable because the patients were involuntarily nervous and worried at the beginning of the training. Thus, the coordination between the patient and robot was very poor. However, once the patient was familiar with the rehabilitation robot, noise points disappear, as reflected in stages 5, 6, and 7. This finding confirmed that it would be necessary for patients to spend some time to familiarize with the machine before the formal training. In the meantime, the special torque_RH displayed different performances from other joints; the reasons could be ascribed to the open-chain structure of the human lower-limb system. Generally, the hip joint acts before the knee joint during human walking. If one side of the lower limb loses its capacity to walk, the hip joint on this side would be useless and unconscious. Thus, the leg and exoskeleton would demonstrate good conformity passively, and it could be surmised that the left leg of the patient was dysfunctional. Upon checking the pathology of the patient, the suspicion had been confirmed. In addition, peak values of both hip and knee torques showed declining trend gradually, and a comparison of the curves showed that the change curves became smoother from the first stage to the last stage. Good trackability brought by the pretraining may be the main reason, but validity of the robot also should be recognized. Therefore, the data on the patient could be used as an index to evaluate the motor ability of the patient, which could help therapists to determine the required diagnostic and therapeutic processes for a patient.

## 4. Conclusions

In response, few lower limb rehabilitation robots had been used in practical health care and huge numbers of patients with lower limb dysfunction in China. An open-structure and applicable treadmill gait trainer (robot) was devised, and key components were analyzed and introduced. Then, a functional prototype was developed, and preliminary experiment on the actual use of the prototype by patients was conducted to validate the functionality of the robot. The experiment showed that different patients and stages demonstrated different performances, and results on the trend variations across patients and across stages suggested that the design may lead to a system that could be successful in the treatment of patients with walking disorder in China. Meanwhile, cost of the robot may be reduced because of localization and independent property rights. This study may provide a reference for similar application design. However, further improvement and therapeutic effects need to be evaluated further.

## Figures and Tables

**Table 1:** Parameters of robot.

Name	Parameter
Degree of freedom of exoskeletons	4 DOF
Adjustment range suspension height	0–500 mm
Adjustable speed of suspension height	5 mm/s
Thrust of lift column	3000 N
Height range of patient	160–185 cm
Adjustment range of armrest width	500–800 mm
Angle of armrest opening	0–900
Treadmill width	800 mm
Width of running surface	450 mm
Treadmill height	290 mm
Treadmill speed	≤0.3 mph
Hip motor	Maxon RE50, 200 watts, 24 V, 5680 rpm,10.8 A
Knee motor	Maxon RE40, 150 watts, 24 V, 6930 rpm, 5.77 A
